# Supplementary material for: Baseline factors predicting the need for corneal crosslinking in patients with keratoconus
Source: PLoS One. 2020 Apr 16;15(4):e0231439. doi: 10.1371/journal.pone.0231439 (PMC7162475; doi:10.1371/journal.pone.0231439)
Supplement: S1 Data — (PDF) [file pone.0231439.s001.pdf]

Case No.	Gender	CXL	Age	Atopic Dermatitis	Pentacam Anterior K2(Steep)	Pentacam Posterior K2(Steep)	Pentacam Total K2(Steep)	Pentacam ISV	Pentacam IVA	Pentacam KI	Pentacam CKI	Pentacam IHA	Pentacam IHD	Pentacam Rmin	Pentacam TCK	Central Corneal Thickness	Thinnest Cornea Thickness
1	M	0	35-39	0	47.7	-7.0	46.5	73	0.84	1.18	1.06	8.9	0.104	6.67	2	471	449
2	F	1	30-34	1	57.9	-9.1	56.4	105	0.63	1.30	1.13	6.3	0.067	5.04	3	480	469
3	F	0	25-29	0	54.3	-8.4	55.1	165	1.92	1.53	1.16	96.7	0.309	4.68	3-4	464	425
4	M	0	25-29	0	42.5	-6.1	41.5	70	0.95	1.20	0.97	17.0	0.088	6.76	2	541	520
5	M	0	45-49	0	55.5	-8.6	53.5	98	0.92	1.30	1.05	78.0	0.173	5.60	2-3	377	352
6	F	1	20-24	0	45.8	-6.8	44.1	30	0.24	1.05	1.00	24.7	0.030	7.07	Poss	525	499
7	F	0	50 or older	0	74.1	-11.6	75.0	185	0.69	1.38	1.31	54.3	0.207	3.38	3-4	444	387
8	M	1	15-19	0	44.8	-6.3	43.8	31	0.30	1.02	0.99	3.4	0.019	7.19	-	523	496
9	M	0	15-19	0	42.2	-6.0	41.2	42	0.49	1.11	0.98	16.3	0.042	7.12	1	472	450
10	M	0	25－29	1	45.1	-6.5	43.8	30	0.23	1.08	1.00	4.6	0.018	7.26	-	533	526
11	M	0	15-19	0	68.5	-13.5	65.1	130	0.41	1.26	1.03	74.6	0.094	4.28	3-4	288	273
12	M	0	40-44	0	56.9	-9.0	54.6	96	0.79	1.22	1.10	10.9	0.070	5.44	2-3	467	425
13	M	0	40-44	0	44.0	-6.6	42.7	62	0.81	1.12	1.03	29.5	0.061	6.98	2	493	479
14	M	0	14 or younger	1	55.6	-7.8	54.4	192	2.17	1.60	1.15	15.4	0.255	4.68	3-4	518	461
15	M	1	15-19	0	52.1	-7.9	50.4	91	0.75	1.21	1.13	11.7	0.101	5.93	2-3	479	461
16	M	1	15-19	0	44.8	-6.3	44.1	93	1.26	1.28	0.99	19.4	0.113	6.15	2-3	463	442
17	F	1	20-24	1	51.0	-8.0	49.8	100	1.12	1.31	1.08	62.5	0.117	5.65	3	503	481
18	M	1	25-29	0	50.6	-7.7	48.9	62	0.52	1.15	1.07	14.0	0.030	6.04	2	465	453
19	M	0	35-39	0	48.1	-7.2	47.3	107	1.35	1.32	1.05	84.4	0.138	5.78	3	541	505
20	M	0	25-29	0	50.8	-7.3	50.0	101	1.07	1.29	1.11	50.5	0.092	5.59	3	491	449
21	F	1	15-19	0	53.4	-8.2	52.8	138	1.53	1.31	1.13	79.3	0.178	5.20	3-4	461	436
22	M	0	20-24	0	43.2	-6.4	42.5	118	1.60	1.32	0.97	45.2	0.147	6.07	Post Cornea	516	489
23	M	1	15-19	0	61.7	-10.0	59.3	108	0.31	1.23	1.13	20.2	0.033	4.82	3	427	412
24	M	0	50 or older	0	46.7	-6.6	45.5	55	0.63	1.13	1.01	22.5	0.038	6.67	1-2	522	487
25	M	0	35-39	0	45.0	-6.6	43.5	56	0.61	1.15	1.03	31.4	0.044	7.07	1-2	509	497
26	F	0	20-24	0	52.0	-7.4	50.9	130	1.50	1.33	1.07	65.8	0.200	5.31	3-4	412	369
27	F	1	20-24	0	49.9	-8.0	48.6	114	1.34	1.34	1.12	72.6	0.103	5.83	3	543	521
28	F	0	25-29	0	46.0	-7.3	44.5	84	1.10	1.24	1.05	6.4	0.101	6.40	2	463	443
29	F	1	15-19	0	72.2	-12.2	69.4	187	0.52	1.43	1.19	7.5	0.085	4.17	3-4	446	384
30	M	1	25-29	0	51.1	-7.9	50.3	109	1.19	1.31	1.10	13.6	0.133	5.48	3	544	499
31	M	0	25-29	0	57.8	-9.1	55.6	120	1.02	1.25	1.12	16.0	0.098	5.30	3	490	414
32	F	0	14 or younger	0	47.5	-7.0	46.0	31	0.24	1.08	1.02	5.4	0.009	6.87	1	503	498
33	F	0	40-44	0	54.4	-8.8	51.8	76	0.38	1.17	1.09	10.0	0.050	5.87	2	498	464
34	F	1	25-29	1	58.0	-9.4	55.8	108	0.80	1.21	1.12	36.4	0.109	4.96	3	446	384
35	F	0	20-24	1	69.3	-11.4	68.0	197	1.22	1.64	1.26	13.3	0.213	4.15	3-4	413	350
36	F	0	20-24	0	45.2	-6.6	44.0	52	0.65	1.12	1.00	30.3	0.060	6.70	1-2	484	465
37	F	0	25-29	0	45.1	-6.5	43.6	25	0.14	1.03	1.00	11.9	0.013	7.29	-	538	534
38	M	0	30-34	0	66.5	-8.8	66.0	248	2.19	2.06	1.20	72.6	0.334	4.27	4	522	388
39	M	1	20-24	0	45.0	-7.3	43.2	37	0.29	1.08	1.05	7.9	0.015	6.85	1	543	535
40	M	0	25-29	1	47.2	-6.8	45.8	118	1.59	1.36	1.03	28.9	0.134	6.34	3	521	477
41	F	0	20-24	0	45.8	-7.0	44.3	57	0.62	1.15	1.02	37.5	0.064	6.65	1-2	490	473
42	M	0	35-39	0	44.8	-6.6	43.7	77	0.96	1.20	1.00	44.0	0.115	6.53	2	455	433
43	M	1	15-19	0	53.8	-8.3	52.6	103	0.89	1.27	1.09	13.2	0.106	5.21	3	501	433
44	M	1	15-19	0	59.6	-8.7	58.8	190	1.70	1.45	1.25	12.1	0.167	4.76	3-4	463	391
45	M	1	25-29	0	58.0	-9.1	57.1	152	1.46	1.49	1.16	4.9	0.168	4.65	3-4	505	391
46	M	1	15-19	0	52.1	-7.6	52.7	166	2.05	1.55	1.09	76.1	0.239	4.92	3-4	504	436
47	M	0	35-39	0	47.2	-7.1	46.1	113	1.45	1.34	1.06	37.6	0.135	6.00	3	467	441
48	M	0	35-39	0	47.1	-7.0	45.9	150	1.98	1.44	1.08	2.9	0.193	5.76	3-4	498	476
49	F	1	15-19	0	53.0	-7.7	53.3	147	1.76	1.44	1.09	116.5	0.243	5.04	3-4	482	448
50	M	0	20-24	0	44.4	-6.5	43.0	31	0.30	1.05	1.02	7.2	0.015	7.39	-	484	471
51	M	1	15-19	0	76.5	-10.7	75.4	260	2.00	2.06	1.30	174.6	0.363	3.57	4	479	361
52	M	1	30-34	1	44.5	-6.3	43.5	70	0.93	1.22	1.00	17.7	0.089	6.50	2	520	478
53	M	0	25-29	0	45.9	-6.7	44.7	40	0.41	1.08	1.02	8.0	0.026	7.16	1	501	485
54	M	1	25-29	0	52.8	-8.1	53.1	199	2.33	1.60	1.14	83.7	0.280	4.46	3-4	502	432
55	F	0	14 or younger	0	46.4	-6.8	44.9	46	0.22	1.08	1.01	2.8	0.006	7.19	1-2	550	546
56	M	1	15-19	0	44.1	-6.4	42.7	46	0.53	1.12	0.98	37.6	0.085	6.76	1-2	527	487
57	M	0	35-39	0	55.7	-8.8	53.9	122	1.21	1.18	1.11	35.2	0.185	5.44	3	489	432
58	F	0	45-49	1	75.3	-12.5	72.3	201	1.01	1.62	1.17	48.9	0.285	4.07	4	374	318
59	M	1	20-24	0	48.2	-7.6	47.3	117	1.57	1.35	1.05	42.2	0.206	5.72	3	534	521
60	M	0	30-34	0	71.5	-11.0	70.4	227	1.85	1.92	1.23	6.3	0.449	4.00	4	478	382
61	M	0	30-34	0	51.5	-8.1	49.5	62	0.50	1.14	1.05	63.3	0.074	6.11	2	495	474
62	M	0	20-24	0	50.6	-7.4	49.4	83	0.89	1.24	1.04	90.9	0.128	5.93	2	452	435
63	M	0	20-24	0	42.9	-6.0	42.6	104	1.46	1.31	1.01	63.3	0.163	6.42	3	561	518
64	M	1	20-24	0	51.1	-7.5	50.1	80	0.70	1.22	1.05	64.4	0.130	5.67	2	490	456
65	F	1	25-29	0	50.1	-7.5	49.2	86	0.79	1.19	1.13	26.1	0.123	5.72	2-3	442	427
66	M	0	15-19	0	44.6	-6.3	43.4	52	0.67	1.13	0.98	31.9	0.081	6.78	1-2	506	486
67	M	0	30-34	0	59.2	-8.7	58.3	137	1.09	1.41	1.16	24.9	0.164	4.97	3-4	460	409
68	M	0	15-19	0	44.2	-6.5	42.7	20	0.24	1.06	1.00	16.2	0.021	7.47	Poss	524	520
69	M	0	45-49	0	47.4	-7.3	46.1	93	1.21	1.28	1.04	118.0	0.161	6.05	2-3	507	468
70	M	1	20-24	0	51.9	-7.6	50.6	117	1.19	1.25	1.13	4.5	0.174	5.50	3	495	457
71	M	1	15-19	0	43.6	-6.7	42.4	84	1.10	1.25	0.99	14.2	0.138	6.29	2	521	497
72	F	0	15-19	0	47.0	-6.7	45.6	48	0.31	1.09	1.01	2.1	0.028	7.08	1-2	541	535
73	M	0	35-39	0	44.2	-6.7	43.1	94	1.28	1.27	1.01	41.3	0.143	6.60	2-3	516	498
74	M	0	25-29	0	50.7	-7.8	50.1	115	1.36	1.35	1.08	64.4	0.196	5.52	3	525	494
75	F	0	14 or younger	0	47.0	-7.2	45.3	39	0.31	1.08	1.01	28.5	0.030	7.02	1	512	505
76	M	0	14 or younger	0	43.4	-6.4	41.9	42	0.51	1.13	1.01	39.9	0.056	7.28	1	537	428
77	M	0	35-39	0	56.6	-8.6	56.7	172	1.81	1.57	1.19	2.5	0.340	4.63	3-4	479	420
78	M	0	35-39	0	51.5	-8.1	49.4	86	0.63	1.24	1.08	59.7	0.084	6.21	2-3	462	448
79	M	1	25-29	0	56.4	-8.9	56.8	177	1.98	1.63	1.15	124.0	0.334	4.71	3-4	505	450
80	M	1	20-24	0	63.4	-9.5	62.8	179	1.70	1.54	1.24	128.2	0.328	4.27	3-4	455	400
81	F	1	14 or younger	0	44.1	-6.3	43.0	45	0.56	1.14	1.00	47.1	0.056	7.33	1	527	520
82	M	0	40-44	1	93.7	-16.2	88.9	222	0.44	1.56	1.29	60.1	0.139	2.82	4	355	276
83	M	0	14 or younger	0	45.1	-7.3	43.1	29	0.26	1.06	1.03	21.1	0.033	7.17	1	545	532
84	M	0	20-24	0	44.7	-7.0	43.1	34	0.37	1.04	1.01	17.7	0.037	7.26	-	558	550
85	M	1	15-19	0	49.2	-7.5	48.8	125	1.54	1.34	1.09	69.2	0.208	5.49	3	473	452
86	M	0	15-19	0	52.1	-9.7	50.0	163	1.49	1.60	1.00	92.8	0.091	4.76	3-4	387	367
87	F	0	15-19	1	50.0	-8.2	47.7	47	0.45	1.11	1.04	52.0	0.066	6.47	1-2	531	503
88	F	1	20-24	0	50.0	-7.8	49.1	85	0.96	1.18	1.08	57.4	0.126	5.63	2-3	451	436
89	M	1	15-19	0	46.5	-6.7	45.6	66	0.81	1.16	1.04	1.7	0.096	6.53	2	550	521
90	M	0	35-39	0	44.5	-6.2	43.5	22	0.20	1.07	1.00	15.5	0.018	7.39	-	520	508
91	M	0	25-29	0	42.4	-6.2	41.4	39	0.49	1.08	0.98	12.9	0.060	7.21	1	556	532
92	M	1	25-29	0	52.0	-7.4	50.9	136	1.61	1.37	1.08	45.7	0.216	5.59	3-4	492	476
93	M	1	15-19	1	77.1	-12.2	75.9	209	1.10	1.51	1.33	67.9	0.315	3.37	4	462	341
94	M	0	50 or older	0	52.1	-8.5	49.7	81	0.65	1.20	1.03	24.3	0.051	5.86	2	464	422
95	F	0	35-39	0	47.2	-6.7	45.7	115	1.45	1.32	0.98	3.2	0.132	6.30	3	501	437
96	M	0	20-24	0	39.2	-5.5	38.1	66	0.83	1.12	0.95	27.3	0.059	7.23	2	555	517
97	M	1	30-34	0	50.4	-7.2	49.3	118	1.50	1.31	1.06	25.9	0.191	6.04	3	515	491
98	M	1	20-24	0	51.7	-8.0	50.1	110	1.22	1.33	1.11	57.5	0.193	5.73	3	478	437
99	M	1	20-24	0	50.6	-6.8	49.5	148	1.89	1.45	1.03	10.7	0.229	5.84	3-4	484	462
100	M	0	35-39	0	76.3	-12.4	73.7	237	1.61	1.72	1.29	74.4	0.421	3.76	4	423	307
101	F	1	20-24	0	53.3	-8.1	52.4	119	1.11	1.27	1.19	67.1	0.170	5.19	3	441	423

102	M	1	15-19	1	61.4	-10.0	57.5	112	0.67	1.33	1.10	16.7	0.008	5.17	3	485	420
103	F	0	50 or older	0	52.0	-7.7	50.4	65	0.61	1.18	1.05	15.3	0.081	6.27	2	440	416
104	M	0	35-39	0	67.8	-11.2	63.9	118	0.29	1.25	1.15	5.6	0.031	4.82	3	396	374
105	M	0	15-19	0	47.7	-7.5	46.2	75	0.84	1.20	1.08	10.7	0.106	6.22	2	490	478
106	M	0	30-34	0	55.2	-8.8	53.1	75	0.59	1.22	1.03	17.3	0.039	5.29	2	420	309
107	M	0	40-44	0	72.3	-11.4	71.2	193	1.24	1.62	1.29	7.6	0.307	3.73	3-4	491	352
108	F	0	50 or older	0	47.5	-6.7	46.6	98	1.23	1.21	0.99	45.0	0.175	5.90	2-3	518	480
109	M	1	15-19	0	59.9	-9.7	59.1	183	1.92	1.65	1.18	68.0	0.355	4.54	3-4	501	411
110	M	1	25-29	0	59.9	-9.3	57.8	92	0.63	1.27	1.03	11.8	0.026	5.03	2-3	368	313
111	M	1	40-44	0	57.5	-9.3	55.5	115	0.78	1.28	1.09	70.9	0.136	5.04	3	529	469
112	M	0	25-29	0	62.1	-10.6	57.3	94	0.82	1.34	0.99	38.3	0.186	4.75	2-3	393	249
113	M	0	25-29	0	65.6	-10.1	65.9	196	1.65	1.55	1.34	180.3	0.363	3.81	3-4	516	451
114	M	0	15-19	1	47.6	-7.2	46.1	30	0.19	1.05	1.03	8.7	0.033	6.87	Poss	501	495
115	F	0	35-39	0	54.0	-8.3	52.3	82	0.78	1.24	1.08	13.3	0.134	5.58	2	467	437
116	M	0	20-24	0	46.5	-7.3	44.6	37	0.42	1.10	1.02	26.8	0.051	6.79	1	535	522
117	F	0	50 or older	0	49.2	-7.4	47.4	26	0.19	1.04	1.02	12.6	0.023	6.64	Abnorm	575	565
118	F	0	50 or older	0	98.6	-16.8	98.6	299	1.26	1.77	1.56	189.6	0.495	2.61	4	390	354
119	M	0	20-24	0	47.6	-7.2	46.6	92	1.13	1.25	1.04	80.1	0.145	6.16	2-3	479	460
120	M	0	20-24	0	60.2	-9.7	59.0	120	0.64	1.25	1.21	87.5	0.105	4.76	3	459	425
121	F	0	40-44	0	49.7	-7.6	48.0	69	0.77	1.11	1.03	19.2	0.093	6.37	2	509	477
122	M	1	14 or younger	0	49.2	-7.0	48.3	160	2.10	1.46	1.04	46.1	0.262	5.64	3-4	473	435
123	M	1	15-19	0	55.1	-8.2	53.5	94	0.76	1.25	1.10	37.4	0.115	5.49	2-3	482	467
124	M	0	35-39	0	44.6	-6.8	43.4	82	1.13	1.24	1.05	53.9	0.130	6.64	2	559	553
125	M	1	20-24	1	72.8	-12.1	69.8	189	0.67	1.44	1.19	8.5	0.170	4.16	3-4	348	269
126	M	0	35-39	0	75.6	-12.5	72.2	197	0.82	1.56	1.19	81.5	0.190	4.23	3-4	402	377
127	M	0	25-29	1	44.9	-6.9	43.0	47	0.51	1.08	0.97	12.0	0.070	6.69	1-2	601	486
128	F	0	35-39	0	77.5	-14.3	74.7	216	0.72	1.11	1.37	44.6	0.039	6.01	4	351	277
129	M	0	30-34	0	46.0	-6.9	44.5	42	0.40	1.08	1.02	37.3	0.047	7.00	1	521	506
130	F	0	25-29	0	49.7	-7.7	48.2	120	1.46	1.38	1.06	25.9	0.207	5.74	3	473	449
131	M	0	20-24	0	49.9	-7.8	49.3	146	1.85	1.50	1.09	14.6	0.257	5.43	3-4	490	439
132	M	1	15-19	1	59.5	-9.2	60.1	222	2.30	1.87	1.25	18.2	0.446	4.47	4	528	439
133	F	1	15-19	0	57.7	-9.0	55.2	164	1.83	1.42	1.18	18.8	0.282	4.81	3-4	445	440
134	F	1	20-24	1	52.7	-8.9	51.7	159	1.83	1.48	1.09	10.4	0.312	4.90	3-4	414	381
135	M	0	20-24	0	42.3	-6.2	41.0	45	0.62	1.12	1.01	38.7	0.069	7.29	1	515	493
136	M	1	35-39	1	55.7	-8.9	53.4	192	2.22	1.67	1.16	9.9	0.295	5.35	3-4	470	393
137	F	1	14 or younger	1	51.2	-7.8	50.3	66	0.51	1.16	1.09	22.6	0.038	5.52	2	454	445
138	M	1	15-19	0	57.7	-8.8	57.5	160	1.60	1.46	1.21	68.7	0.305	4.70	3-4	493	440
139	F	0	14 or younger	0	46.8	-6.9	45.5	31	0.14	1.05	1.04	2.9	0.009	6.89	1	511	503
140	M	1	15-19	1	72.2	-11.0	70.6	180	0.67	1.47	1.24	53.2	0.151	4.29	3-4	415	385
141	M	1	30-34	0	47.5	-6.6	46.4	113	1.50	1.32	0.99	18.5	0.173	6.12	3	509	481
142	M	1	20-24	1	72.8	-10.9	68.1	219	1.39	1.64	1.23	62.6	0.324	3.99	4	450	343
143	M	0	20-24	0	52.6	-7.9	51.2	115	1.27	1.36	1.11	25.2	0.186	5.58	3	454	399
144	M	0	30-34	0	59.8	-9.8	57.7	110	0.45	1.24	1.19	31.6	0.075	4.79	3	454	436
145	F	0	20-24	0	51.7	-8.4	49.6	88	0.76	1.21	1.11	12.3	0.102	5.91	2-3	479	451
146	F	0	30-34	0	50.3	-7.5	49.5	93	1.03	1.25	1.09	89.0	0.147	5.81	2-3	456	432
147	M	1	15-19	0	42.4	-6.2	41.4	77	1.08	1.19	0.98	22.4	0.119	6.77	2	504	481
148	F	0	20-24	0	50.5	-7.8	50.0	122	1.40	1.40	1.11	57.0	0.208	5.38	3	536	514
149	M	0	20-24	0	48.7	-7.2	47.3	68	0.65	1.15	1.07	3.7	0.085	6.48	2	464	451
150	F	0	50 or older	0	57.9	-9.9	55.2	112	0.96	1.34	1.04	41.5	0.128	5.09	3	434	367
151	M	0	20-24	0	45.0	-6.7	43.5	61.00	0.76	1.14	1.0	14.20	0.10	6.47	2	543	500
152	M	0	40-44	0	55.2	-8.1	54.1	109	0.78	1.25	1.17	12.5	0.073	5.04	3	473	449
153	M	0	45-49	0	73.0	-12.4	69.8	203	0.89	1.44	1.27	27.3	0.224	4.06	4	344	314
154	F	0	45-49	0	59.2	-9.2	56.9	159	1.56	1.30	1.16	29.9	0.183	4.93	3-4	482	449
155	M	0	40-44	0	47.7	-7.4	46.4	103	1.34	1.34	1.04	58.1	0.173	6.07	3	474	446
156	F	0	50 or older	0	56.2	-8.8	54.6	107	0.89	1.23	1.14	42.8	0.139	5.30	3	479	472
157	M	0	30-34	0	48.6	-7.4	46.8	83	0.97	1.21	1.04	26.1	0.084	6.59	2	495	450
158	M	1	35-39	0	77.6	-13.3	73.1	204	1.10	1.65	1.25	104.8	0.230	3.88	4	460	320
